# Illustrations of the Sedative and Febrifuge Powers of Emetic Tartar

**Published:** 1824-04

**Authors:** William Balfour

**Affiliations:** the University of Edinburgh.


**Art. III.**—*Illustrations of the Sedative and Febrifuge Powers of Emetic Tartar.* By William Balfour, M.D. of the University of Edinburgh.

Ne quid falsi dicere audeam, ne quid veri non audeam.

The progress of improvement in the science of medicine has, in all ages, been extremely slow. From the beginning of time to the present day, truths have been latent, which, when once brought to light, every one has been surprised he himself did not discover. It is the best test, indeed, of the reality and importance of any improvement, that, to command assent, it requires only to be proposed.

It cannot be denied that, in the treatment of inflammation, blood-letting is often practised to an extent, which renders it difficult to say whether the patient is in most danger from the disease or from the means of cure. Reflecting, some years ago, on this subject, it appeared to me that the discovery of a remedy calculated to supersede the necessity of carrying depletion such dangerous lengths, was one of the most important desiderata in the practice of medicine. I had not long directed my attention to the inquiry, when emetic tartar presented itself to my mind, as the substance most likely to accomplish the object in view. From the effects which it has been long known to produce on the exhalents of the skin, it appeared to me probable that it exerted a similar power on all the exhalents of the body: consequently, that it must relax, and render pervious, not only the exhalents themselves, but all those vessels, also, of which they are a continuation. Now, as, in all inflammatory complaints, the functions of secretion and excretion are suspended or impaired in proportion to the degree of inflammation present, the restoration and support of these functions is the change to be effected in the treatment of inflammation. In other words, to restore these functions is to effect a cure. Therefore I concluded that, if emetic tartar influenced the other exhalents of the body equally with those of the skin, it must prove a powerful auxiliary to blood-letting, in the cure of inflammation. I subjected it to experiment, and obtained results, the benefit of which to mankind, the facts I have already laid before the public,* as well as those which follow, will attest. As often happens in experiments and observations instituted with a particular view, I made a more important discovery than I had anticipated. I little expected to find the medicine in question to be a direct sedative, independently of its nauseating and sudorific qualities.

Master Henry Ormston, aged fifteen, was seized with pain immediately under the fifth and sixth ribs of the left side, on the evening of the 31st of January, 1820. The pain continued all night, and increased in the course of next day to an alarming height. It was confined to one spot, for the most part, but at times shot as high as the clavicle. I was called on the 1st of February, at half-past four o'clock P.M., and found the boy sitting at an open window for air, as he could not breathe at all in the recumbent posture. Respiration was performed, indeed, with the greatest difficulty; his countenance was pale as death, and his lips alternately pale and livid; pulse 80, small, and oppressed; bowels obstinately costive. I ordered the patient to bed immediately, took eight ounces of blood from his arm, and administered a fourth of a grain of emetic tartar in solution; directing the same quantity to be taken every hour.

Half-past six o'clock.—Had no relief for half an hour, when he came to breathe easier. Medicine remained on the stomach. Has had a second dose, which has also been retained. Feels a tendency to sickness; pulse 100, and full; skin hot, and dry; has complained of cold, and has had some rigors.

Ten o'clock.—Vomited copiously, but easily, immediately after last visit; when the chilliness went off, and he had no pain for two hours. It returned, however, at half-past nine, and is as severe, the patient thinks, as ever it was. Has had the third dose of the medicine, about ten minutes ago. It had been improperly intermitted on account of the vomiting. Pulse 100, full and percutient; skin hot and dry, with considerable thirst.

Satisfied that the freedom from pain for two hours, which the patient experienced, was to be attributed chiefly to the sedative powers of the medicine, I considered myself warranted to wait its effects a second time. Accordingly, I remained with him half an hour, and had the satisfaction of observing his breathing become gradually easier. I ordered an ounce of Epsom salts to be given in divided doses, but all within half an hour; the antimonial to be continued every hour through the night, unless the patient should happily be invaded by sleep; a blistering plaster to be in readiness, and applied to the part affected at twelve o'clock, if the pain continued troublesome; and that I

* See *Illustrations of the Sedative and Febrifuge Powers of Emetic Tartar*, 1818 and 1819.

should be called at any hour of the night, on the slightest appearance of the symptoms becoming worse.

February 2d, eleven o'clock A.M.—Had a very tolerable night, with some sleep, and a breathing sweat of some hours. The vesicatory was not applied. Pulse 80, full and soft; breathing easy; no pain in the side, except upon motion; bowels unmoved; no sickness or vomiting from the medicine. Intermit the antimonial. To a decoction of half an ounce of senna in eight ounces of water, add an ounce of Epsom salts, and give a wine-glassful every hour, till the bowels are sufficiently cleared.

Seven o'clock P.M.—The purgative has operated five times. Pulse 92, and percutient; pain in the side increased; effects attributable entirely to want of caution when the patient had occasion to rise. Resume the antimonial as before.

February 3d, eleven o'clock A.M.—Pain in the side became easier soon after beginning the antimonial again. Pulse 80, and soft. Continue the antimonial every two or three hours; and the decoction as occasion requires.

Nine o'clock P.M.—Has no pain, except on attempting to lie on the side affected. Continue the antimonial.

February 4th.—Slept perfectly well through the night, but thinks he cannot yet lie on the left side. Continue the antimonial.

February 5th.—Had a good night. Found himself, in the morning, on his left side; and, on the 7th, went abroad.

Thus, this severe case of pleurisy was cured, *in three days*, by the detraction of only eight ounces of blood, the exhibition of eight grains of emetic tartar, and a purgative. The emetic tartar occasioned no nausea or vomiting after the second dose.

Some time ago, a very near relative of my own, also fifteen years of age, was seized with pneumonia. I committed him to Dr. Gregory and an eminent surgeon. They took about seventy ounces of blood from him, in three or four days. Except purgatives, indeed, and the inhalation of steam, he had no other remedy. He suddenly became extremely exhausted. The family were collected at his bedside, one morning at six o'clock, to see him die. He recruited a little till eleven, when he appeared at the last gasp. The cuticle came off his breast, on the finger being drawn slightly across. In despair, I gave him wine, which was strictly prohibited by Dr. Gregory the night before, and saved him.

Let it not be inferred, however, that I was dissatisfied with the conduct of these two gentlemen, or that I mention these facts now in the spirit of detraction: on the contrary, nothing could exceed their sympathy and attention, which I shall ever remember with the liveliest gratitude. My only motive, therefore, for recurring to the case is to show that the most eminent practitioners in Edinburgh were, at that time, strangers to the sedative and febrifuge powers of emetic tartar. Had it been otherwise, would not Dr. Gregory have employed it in the case alluded to, in aid of the lancet?

On the 24th of June, 1817, I was called to Mrs. Lawson, aged thirty-five, whom I found labouring under pleuritic inflammation, of great severity, in the right side. The pain was most acute in one particular spot, but extended a great way in every direction. She had been delivered of a child a month before. I immediately bled her to sixteen ounces, when faintness, approaching to *animi deliquium*, prevented me going farther. The pain, however, was greatly diminished; but inflammatory action recurred next day, in all its former violence, and yielded to the same treatment. This happened repeatedly afterwards; so that, independently of blistering, purging, and calomel in alterative doses, she lost about eighty ounces of blood in six days, and at five bleedings.

Convinced that blood-letting, if carried much farther, would infallibly kill, but could not cure, my patient, I requested a consultation with Dr. Hamilton, professor of midwifery in this university. From the rigors, combined with other circumstances, he strongly suspected suppuration had taken place: that was his only reason for not carrying blood-letting farther. He advised digitalis in small doses; failing which, tobacco injections, in order to induce nausea and general relaxation. She got the digitalis; and, of my own judgment, I afterwards exhibited Dover's powder, with the view of determining to the surface. Blistering was also repeated; and the woman recovered.

On this occasion, though the Professor, previous to giving an opinion, inquired minutely into every step I had taken, yet he made no mention of emetic tartar, or of any of the preparations of antimony. It follows that, at that time, he was equally unacquainted as myself with the medicinal powers of emetic tartar in inflammation. Had he known it to be, or believed it, capable of producing such effects as I have detailed in Master Ormston's case, and elsewhere, he would have regretted I had not employed it;* and must certainly have prescribed it, instead of infusion of tobacco, for the purpose of inducing nausea and general relaxation.

I again protest, that it is not for the purpose of censuring I allude to the practice of this or any other gentleman: my only object is the vindication of my just claim to originality, in what

---

* He regretted I had not used the remedy which, he said, *he had introduced into practice*,—viz. infusion of tobacco.

I have advanced concerning the sedative and febrifuge powers of emetic tartar.

This claim has excited the sneers of some, who will allow no merit to a contemporary practising on the same spot with themselves. I never had an opportunity, however, of grappling, on the subject, with such opponents, without reducing them to the vague and general argument, "that tartrite of antimony and potass has been employed ever since chemical medicines came into use."*

Now, by parity of reasoning, it might be affirmed that Harvey did not discover the circulation of the blood, because many observations were made before his time, which paved the way for, and nearly approached to, the grand discovery which has immortalised his name?† By parity of reasoning, it might be

---

* Before I published (in 1818) on Emetic Tartar, I consulted several practitioners, of the highest eminence, concerning their experience on the subject, and found them, to a man, total strangers to the administration of the medicine, *qua* sedative and febrifuge. Dr. Hamilton, sen. author of a Treatise on Purgative Medicines, and a physician inferior to none of his day, frankly told me he had not used emetic tartar at all, for a number of years. The fact is, Dr. Cullen frightened the practitioners of Edinburgh from the use of it, by his always administering and recommending it in nauseating doses, in the erroneous belief that in smaller doses it was inefficient.

† Galen observed, and has described, the motion of the blood, or animal spirits, through the lungs into the left side of the heart;* and also the anastomoses of the veins and arteries.† Erasistratus‡ made great advances to the discovery of the circulation, in restraining hemorrhagy in both superior and inferior extremities, by ligatures. Andreas Vesalius not only taught that the septum of the heart is impervious, but that, in certain circumstances, the blood passed from the branches into the trunks of the veins; an observation still nearer the truth. G. Fallopius|| found that an artery does not pulsate beyond a ligature; and, therefore, inferred that pulsation is not an inherent power in arteries. Is it not astonishing he did not inquire what the power, the *vis a tergo*, was that produced the dilatation? Realdus Columbus¶ established the doctrine of the lesser circulation, from the argument taken from the size of the pulmonary artery, and from the blood he found in the left side of the heart; where, according to Galen, animal spirits only should have been. The doctrine of the lesser circulation was known, indeed, to many before Harvey's time. Julius Cæsar Arantius** was sufficiently aware of it; and not only denied the existence of any communication between the right and left side, through the septum of the heart, but demonstrated the great size of the pulmonary artery; and also the situation and functions of the valves in the left side, which, he maintained, admitted the blood in its progress from the lungs, but precluded its return. Fabricius ab Aquapendente discovered the valves in the veins, but was ignorant of their use. How nearly did these anatomists approach the important fact! Had any of them gone but a single step farther, the honour of the discovery would have been his. It was reserved for Harvey, however, "to go the little farther;" and but a little farther than his predecessors did he go, when he made the most important discovery that ever was, or ever will be, made by man. All, or almost all, the necessary facts were furnished to his hand; and the wonder is, not that he drew the conclusion, but that the important truth should, for so many ages, have escaped observation.

---

* *De Usu Partium.* † *De Utilat. Puls.*

‡ Celsus *de Corp. Human. Fabric.*

|| *De Part. Simil.*

¶ *De Re Anatomica.* ** *Observat. Anatomie.*

asserted that Dr. Jenner, in propagating cow-pox by inoculation, conferred no benefit on mankind; because it must have been known, as well to medical practitioners as to the common people, ages before he was born, that vaccine matter, received into the human body, rendered it proof against the variolous infection. But, till Jenner arose, no one availed himself of such knowledge,—no one reasoned a moment on the subject. Now, however, that advantage is taken of the fact, nothing appears so easy, nothing so natural. Our astonishment is, not that the subject arrested Dr. Jenner's attention, but that it should have been so long entirely overlooked. By parity of reasoning, it might be affirmed that Dr. Hamilton has thrown no light on the administration and efficacy of purgative medicines, since purgative medicines have been in use from the earliest dawn of medical science. But Dr. Hamilton is, and must for ever be, considered as having made one of the most important practical improvements that medicine has received in modern times.

The candid reader will not fail to appreciate the contrast between the preceding summary and the following case:

On the 22d of February, 1820, I was called to the same woman (Mrs. Lawson), and found her in circumstances similar to those already detailed. There was this difference, that she was now very stout, having had no children, and having enjoyed good health since her former illness, about two years and a half before. On the 21st, it blew a tremendous gale from the north-east, accompanied with rain and intense cold. To these she had been exposed in the evening, and was soon after seized with pain in the right side, at the pit of the stomach, and in the abdomen. These symptoms increased in the course of the night and next day, to an unbearable pitch. I saw her at half-past three P.M., and never witnessed a person in greater distress. Fixed pain under the fifth and sixth ribs, extending to the back, to the pit of the stomach, and to the abdomen, rendered respiration extremely difficult and painful. She often cried, "I am all on fire, and can neither breathe nor lie in any posture." The stomach and abdomen were extremely tender to the touch; great thirst; pulse 80, small and irregular; frequent rigors, which shook the whole room.

I instantly gave her a grain of emetic tartar in solution, and took eighteen ounces of blood from her arm. For half an hour she had no relief: she then became sick, and, in a quarter of an hour after, vomited copiously, but easily, or without increase of the local pain. Shortly after, half a grain of the medicine was administered as before, which was retained till she took another half grain, in about half an hour. The last dose occasioned instant vomiting, but not the slightest distress.

Seven o'clock.—Pain in the side somewhat abated, but intense all over the abdomen, particularly in the umbilical region; frequent rigors; pulse 60, much fuller and softer. Admits she felt relieved for some time before and after vomiting. Apply dry warm flannel to the abdomen; and let her have the medicine in half-grain doses every hour, whether it occasions sickness or not.

Ten o'clock.—Greatly relieved in every respect. The medicine has been retained; and she can lie and breathe without pain, but cannot move without exciting it. Pulse still 60; no rigors since last visit. Continue the medicine through the night, according to the urgency of symptoms.

February 23d.—Passed the night very well; took only one dose of the medicine; lies, moves in every direction, and breathes with perfect freedom. Pulse 62; bowels costive. Take a dose of senna and Epsom salts.

February 26th.—No return of pain; and the patient is going about her household affairs.

In the case treated by Dr. Gregory, and in Mrs. Lawson's first illness, the ordinary practice was followed,—that is, blood-letting was trusted to, almost exclusively;* yet, in both cases, the cure was tedious and difficult. Nay, Dr. Gregory found it necessary to take so much blood from his patient, that, had I not timely given him wine, he must have died of exhaustion.

On the other hand, in Master Ormston's case, and in Mrs. Lawson's second attack, emetic tartar was, after a moderate bleeding, administered steadily and perseveringly, as the chief remedy, without producing nausea, except at first; and, in both cases, the cure was rapid beyond example.

Notwithstanding these, and a multitude of facts of similar import, the reply or observation is, "Tartrite of antimony was known before, and has been in use for centuries." I never denied, but, on the contrary, affirmed,† that it has been long in use, and known as emetic, sudorific, and (in nauseating doses) sedative. But, so far as I know, I first discovered that it is directly and powerfully sedative, independently of its nauseating and evacuant powers. Any substance is sedative, in so far as it is nauseating. The very tendency of evacuation is to lower the action of the system. If, therefore, emetic tartar was not known, before I wrote on the subject, to be sedative, any farther than it was nauseating;—in other words, if it was believed

* Dr. Gregory's aversion to emetics, particularly tartar emetic, is well known. It is equally well known that he gave the *ton* to practice. Now, I do not believe he ever prescribed tartar emetic in his life, except, perhaps, with the view of its being immediately rejected; certainly never with the intention of its being absorbed into the system. Is it to be wondered at, then, that a medicine which he proscribed should be neglected by those who studied under him,—who looked up to him?

† See my *Treatise on the Sedative and Febrifuge Powers of Emetic Tartar*, second Edition, 1819.

that its sedative depended on its nauseating powers, then I deny that it was known at all as a sedative, properly so called.

Medicines are, or ought to be, arranged, not according to their ultimate, but their primary, most simple, and most constant, effects. If tartrite of antimony did not increase the cuticular discharge, except when administered in nauseating and emetic doses, it would have no claim to be denominated sudorific. Tonics and stimulants often prove powerful sedatives in their ultimate effects. But, if it promotes the action of the vessels of the skin, without previously occasioning either nausea or vomiting, then it is, properly speaking, a sudorific. By parity of reasoning, if tartrite of antimony diminishes the action of the system, without previously occasioning nausea, vomiting, or sensible increase of perspiration, that effect cannot depend on these as causes, but is altogether independent of them. But I have adduced innumerable instances of emetic tartar subduing inflammatory action, not by inducing nausea and increase of sensible perspiration, but directly, without the intervention of any sensible effects whatever. It irresistibly follows, that emetic tartar is a powerful sedative, properly so called.

[To be continued.]

---

Art. IV.—*On Medicinal Saturation.* By Dr. Kinglake.

