# Errata in Last Number

**Published:** 1824-05

**Authors:** 


					in uie motto to Dr. Balfour s Paper, for discere read dicere.

				

